# Decreased AdipoR1 signaling and its implications for obesity-induced male infertility

**DOI:** 10.1038/s41598-024-56290-0

**Published:** 2024-03-08

**Authors:** Toshiko Kobori, Masato Iwabu, Miki Okada-Iwabu, Nozomi Ohuchi, Akiko Kikuchi, Naoko Yamauchi, Takashi Kadowaki, Toshimasa Yamauchi, Masato Kasuga

**Affiliations:** 1 Division of Diabetes and Metabolism, The Institute of Medical Science, Asahi Life Foundation, Chuo-Ku, Tokyo, 103-0002 Japan; 2 Department of Endocrinology, Metabolism and Nephrology, Graduate School of Medicine, Nippon Medical School, Bunkyo-Ku, Tokyo, 113-8603 Japan; 3 Department of Diabetes and Metabolic Diseases, Graduate School of Medicine, The University of Tokyo, Bunkyo-Ku, Tokyo, 113-8655 Japan; 4 Laboratory for Advanced Research on Pathophysiology of Metabolic Diseases, The University of Tokyo, Bunkyo-Ku, Tokyo, 113-8655 Japan; 5 Digital Pathology Center, Asahi General Hospital, Asahi-Shi, Chiba, 289-2511 Japan; 6 Toranomon Hospital, Minato-Ku, Tokyo, 105-8470 Japan

**Keywords:** Diseases, Molecular medicine

## Abstract

Obesity is among the risk factors for male infertility. Although several mechanisms underlying obesity-induced male subfertility have been reported, the entire mechanism of obesity-induced male infertility still remains unclear. Here, we show that sperm count, sperm motility and sperm fertilizing ability were decreased in male mice fed a high-fat diet and that the expression of the AdipoR1 gene and protein was decreased, and the expression of pro-apoptotic genes and protein increased, in the testis from mice fed a high-fat diet. Moreover, we demonstrate that testes weight, sperm count, sperm motility and sperm fertilizing ability were significantly decreased in AdipoR1 knockout mice compared to those in wild-type mice; furthermore, the phosphorylation of AMPK was decreased, and the expression of pro-apoptotic genes and proteins, caspase-6 activity and pathologically apoptotic seminiferous tubules were increased, in the testis from AdipoR1 knockout mice. Furthermore, study findings show that orally administrated AdipoRon decreased caspase-6 activity and apoptotic seminiferous tubules in the testis, thus ameliorating sperm motility in male mice fed a high-fat diet. This was the first study to demonstrate that decreased AdipoR1/AMPK signaling led to increased caspase-6 activity/increased apoptosis in the testis thus likely accounting for male infertility.

## Introduction

According to the World Health Organization (WHO), approximately 13% of the world’s adult population were obese in 2016, and the worldwide prevalence of obesity nearly tripled between 1975 and 2016^1^. Obesity is known to cause insulin resistance, which is in turn associated with type 2 diabetes and cardiovascular disease^2–4^, and decreased adiponectin in plasma in obesity are shown to be implicated as a cause of these obesity-linked diseases^5–7^.

Adiponectin^8–11^, a protein secreted from and highly specifically expressed in adipose tissue and known as an adipokine^12–14^, has anti-inflammatory and insulin-sensitizing properties^15^. Adiponectin is shown to be decreased in plasma in obesity, insulin resistance and type 2 diabetes, while adiponectin supplementation is shown to ameliorate insulin resistance and impaired glucose tolerance in mice^16–19^.

We previously reported cloning of AdipoR1 and AdipoR2 as receptors for adiponectin^20^. AdipoR1 and AdipoR2 were each assumed to have a seven-transmembrane topology with an internal N-terminus and an external C-terminus, opposite to that of G-protein-coupled receptors (GPCRs)^20^, and the crystal structures of human AdipoR1 and AdipoR2 are shown to represent a novel class of receptor structures with the seven-transmembrane helices, conformationally distinct from those of GPCRs, shown to enclose a large cavity where three conserved histidine residues coordinating a zinc ion^21,22^. AdipoR1 and AdipoR2 serve as the most physiologically important receptors for adiponectin, with AdipoR1 and AdipoR2 shown to activate the AMPK^23^ and PPAR-α^24^ pathways, respectively^20,25,26^. With its expression shown to be decreased in obesity^27^, similarly to adiponectin, AdipoRs are assumed to play important roles in the regulation of glucose and lipid metabolism, as well as in inflammation and oxidative stress, in vivo^28–30^.

Infertility is a global public health issue affecting 10–15% of couples in reproductive age^31^. Male factors per se account for 25–30% of all cases of infertility but also account for another 30% when combined with female factors. Thus, approximately 50% of infertility is attributable to male factors^32^. While known etiologies of male infertility include cryptorchidism, testicular torsion or trauma, varicocele, seminal tract infections, antisperm antibodies, hypogonadotropic hypogonadism, gonadal dysgenesis, and obstruction of the reproductive channels^32^, obesity has been reported to represent a risk factor for male subfertility^33–36^. Several mechanisms underlying obesity-induced male subfertility have been reported, which include hypogonadism, chronic inflammation, oxidative stress, impaired sperm parameters, such as sperm concentration, sperm motility and morphology, sperm DNA damage, altered sperm lipid composition, and sperm epigenetic modification^37^. However, the entire mechanism of obesity-induced male subfertility remains poorly elucidated.

A previous clinical cross-sectional study reported that serum and seminal plasma adiponectin levels were significantly lower in men with body mass index (BMI) ≥ 25 kg/m^2^ compared to those with BMI < 25 kg/m^2^ and that adiponectin concentration in seminal plasma significantly is positively correlated with sperm parameters, such as sperm concentration, sperm count and total normomorphic spermatozoa^38^. Furthermore, administration of recombinant adiponectin was shown in an in vivo study to ameliorate testicular dysfunction in diabetes model mice induced by high-fat diet and/or streptozotocin^39,40^.

In this study, AdipoR knockout (KO) mice were analyzed to investigate whether decreased adiponectin/AdipoR signaling might be associated with obesity-induced male infertility, and if so, to clarify the mechanism by which decreased adiponectin/AdipoR signaling might induce male infertility.

## Results

### Impaired spermatogenesis and infertility in high-fat-diet-induced obese mice

**Figure 1:**
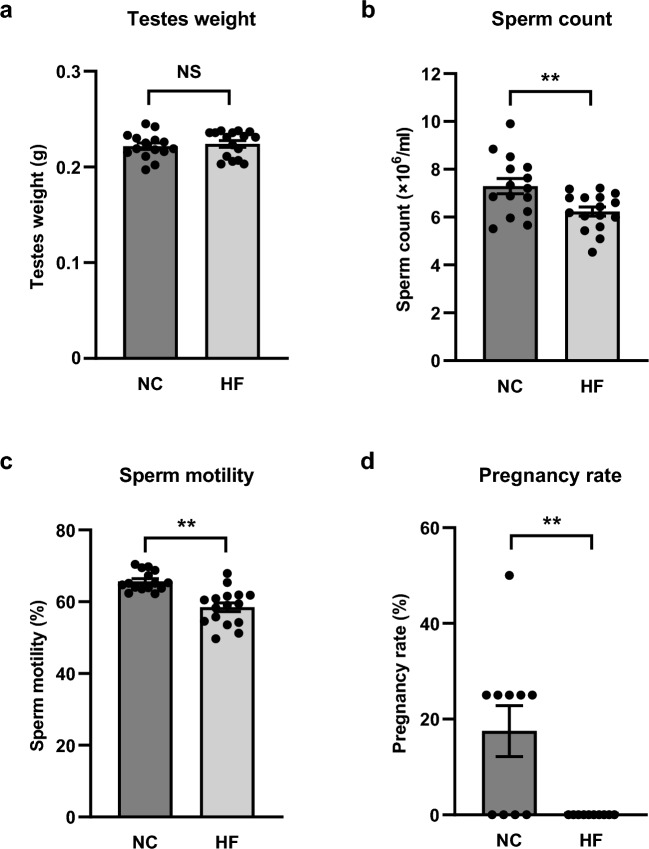
Sperm count, sperm motility and sperm fertilizing ability in male mice fed a high-fat diet were decreased. Testes weight (**a**), sperm count (**b**), and sperm motility (**c**) in mice fed a normal chow diet (NC) and a high-fat diet (HF) for 22–23 weeks. Pregnancy rate in mice fed a NC and a HF for 28 weeks (**d**). All values are presented as means ± s.e.m. **P < 0.01 compared to mice fed a NC. *P* values were determined by the unpaired two-tailed *t*-test. NS, not significant. NC, n = 15 (**a**–**c**), n = 10 (**d**); HF, n = 16 (**a**–**c**), n = 10 (**d**).

In order to investigate the impact of high-fat diet on the fertility of male mice, we analyzed testis and semen from mice fed a normal chow diet or a high-fat diet. Although there was no significant difference in testes weight between the mice fed a normal chow diet and a high-fat diet (Fig. 1a), sperm count and sperm motility were significantly decreased in high-fat diet-induced obese mice (Fig. 1b,c). Next, we assessed male mice for fertility using a mating assay and showed that the pregnancy rate in male mice fed a high-fat diet was significantly lower than that in mice fed a normal chow diet (Fig. 1d). These results suggest that high-fat diet induced male infertility by impairing spermatogenesis. Furthermore, sperm count, sperm motility and sperm fertilizing ability were also significantly decreased in a male mouse model of type 1 diabetes (Fig. S1a–c); it was also suggested that the fertility rate might be more decreased in mice fed a high-fat diet than that in the mouse model of type 1 diabetes (Fig. 1d, Fig. S1c).

### Decreased expression of the AdipoR1, increased expression of pro-apoptotic genes and proteins, and increased TUNEL-positive seminiferous tubules in the testis from mice fed a high-fat diet

**Figure 2:**
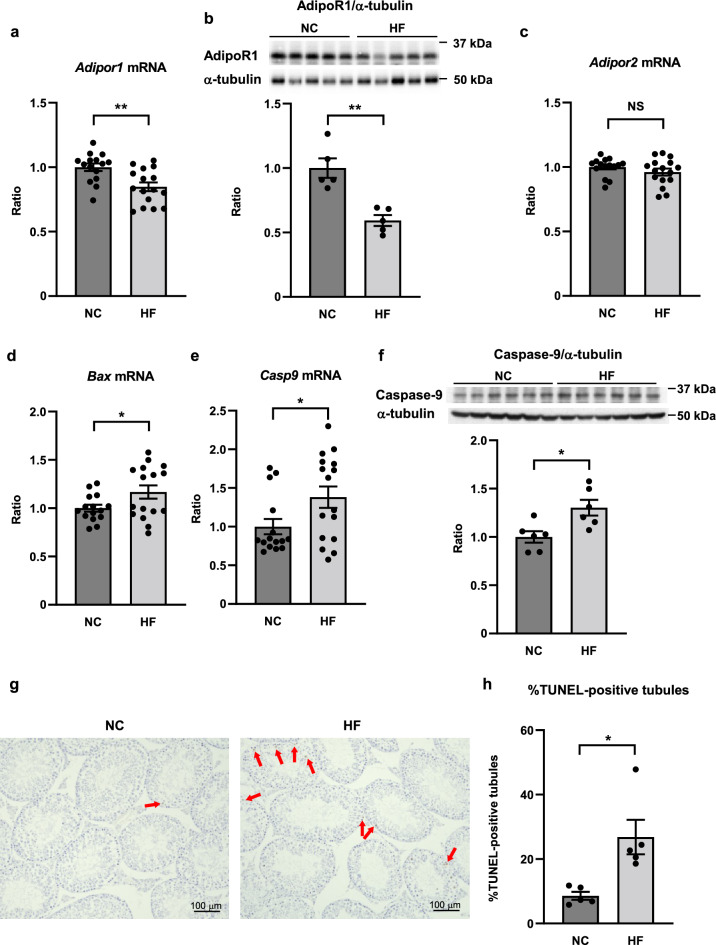
Expression of AdipoR1 was decreased and expression of pro-apoptotic genes, proteins and TUNEL-positive seminiferous tubules were increased in the testis from mice fed a high-fat diet. *Adipor1* (**a**), *Adipor2* (**c**), *Bax* (**d**) and *Casp9* (**e**) mRNA levels, and AdipoR1 (**b**) and caspase-9 (**f**) protein levels in the testis from mice fed a normal chow diet (NC) and a high-fat diet (HF) for 22–23 weeks. Results of real-time PCR were normalized to *Rn18s*. Results of western blot were normalized to α-tubulin. Representative micrographs from TUNEL staining of the testis from mice fed a NC and HF (**g**) and percentage of seminiferous tubules with TUNEL-positive cells in mice fed a NC and HF (**h**). All values are presented as means ± s.e.m. *P < 0.05 and **P < 0.01 compared to mice fed a NC. *P* values were determined by the unpaired two-tailed *t*-test. Scale bar, 100 μm. The red arrows indicate TUNEL-positive cells. NS, not significant. NC, n = 15 (**a, c-e**), n = 5 (**b, h**), n = 6 (**f**); HF, n = 16 (**a, c-e**), n = 5 (**b, h**), n = 6 (**f**).

To clarify the molecular mechanism by which high-fat diet affects male infertility, we performed gene expression and western blot analyses. High-fat diet significantly decreased the expression of AdipoR1 in the testis compared to that in normal chow diet both in the mRNA and the protein levels (Fig. 2a,b) although there was no significant difference in the expression of AdipoR2 (*Adipor2*) (Fig. 2c). Moreover, high-fat diet significantly increased the expression of the pro-apoptotic genes, such as Bcl-2-associated X protein (*Bax*) (Fig. 2d) and caspase-9 (*Casp9*) (Fig. 2e), as well as the expression of caspase-9 at the protein level (Fig. 2f). Furthermore, we went on to evaluate apoptosis in the testis by terminal transferase-mediated deoxyuridine triphosphate nick end labeling (TUNEL) staining and demonstrated that high-fat diet increased the number of apoptotic seminiferous tubules (Fig. 2g,h).

### Impaired spermatogenesis and infertility in AdipoR1 KO mice

**Figure 3:**
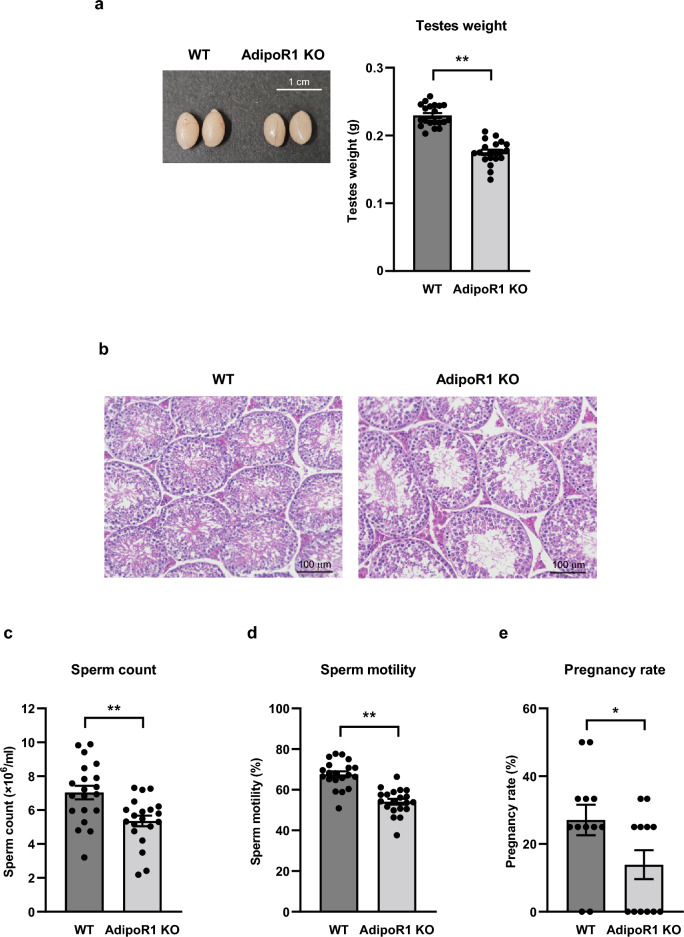
Testes weight, sperm count, sperm motility and sperm fertilizing ability in AdipoR1 KO mice were decreased. A representative macrograph of testes and testes weight (**a**), representative micrographs from hematoxylin and eosin staining of the testis (**b**), sperm count (**c**), sperm motility (**d**) and pregnancy rate (**e**) in wild-type (WT) mice and AdipoR1 knockout (KO) mice. All values are presented as means ± s.e.m. *P < 0.05 and **P < 0.01 compared to WT. *P* values were determined by the unpaired two-tailed *t*-test. Scale bar, 1 cm (**a**), 100 μm (**b**). WT, n = 20 (**a, c, d**), n = 12 (**e**); AdipoR1 KO, n = 20 (**a, c, d**), n = 12 (**e**).

Based on evidence of decreased expression of the AdipoR1 gene in the testis from mice fed a high-fat diet, we next examined male AdipoR1 KO mice for fertility, and demonstrated that, interestingly, testes weight was significantly decreased (Fig. 3a) and seminiferous tubules were atrophied (Fig. 3b) in AdipoR1 KO mice compared to those in wild-type mice. Moreover, sperm count (Fig. 3c), sperm motility (Fig. 3d), and pregnancy rate (Fig. 3e) were significantly decreased in AdipoR1 KO mice compared to those in wild-type mice.

### Increased expression of pro-apoptotic genes and proteins in the testis from AdipoR1 KO mice

**Figure 4:**
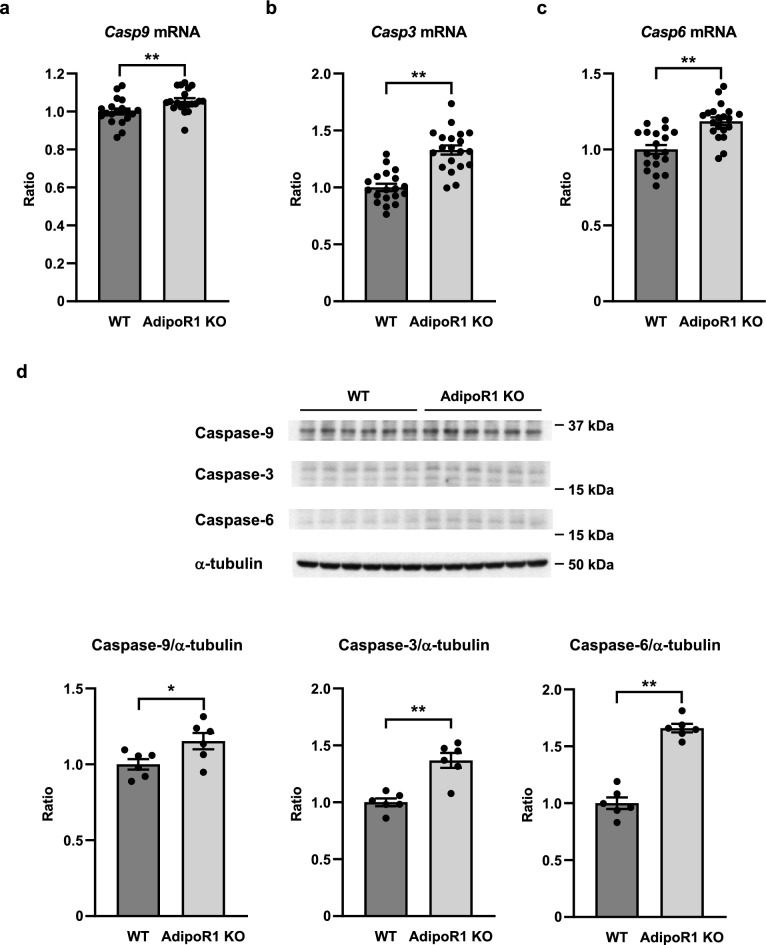
Expression of pro-apoptotic genes and proteins were increased in the testis from AdipoR1 KO mice. *Casp9* (**a**), *Casp3* (**b**) and *Casp6* (**c**) mRNA levels and caspase-9, caspase-3 and caspase-6 protein levels (**d**) in the testis from wild-type (WT) mice and AdipoR1 knockout (KO) mice. Results of real-time PCR were normalized to *Rn18s*. Results of western blot were normalized to α-tubulin. All values are presented as means ± s.e.m. *P < 0.05 and **P < 0.01 compared to WT. *P* values were determined by the unpaired two-tailed *t*-test. WT, n = 19 (**a, b, c**), n = 6 (**d**); AdipoR1 KO, n = 20 (**a, b, c**), n = 6 (**d**).

We next performed gene expression and western blot analyses on the testis from AdipoR1 KO mice and demonstrated that the expression of pro-apoptotic genes and proteins, such as caspase-9 (Fig. 4a,d), caspase-3 (Fig. 4b,d) and caspase-6 (Fig. 4c,d) were significantly increased in the testis from AdipoR1 KO mice compared to that in wild-type mice.

### Decreased phosphorylation of AMPK and increased apoptosis in the testis from AdipoR1 KO mice

**Figure 5:**
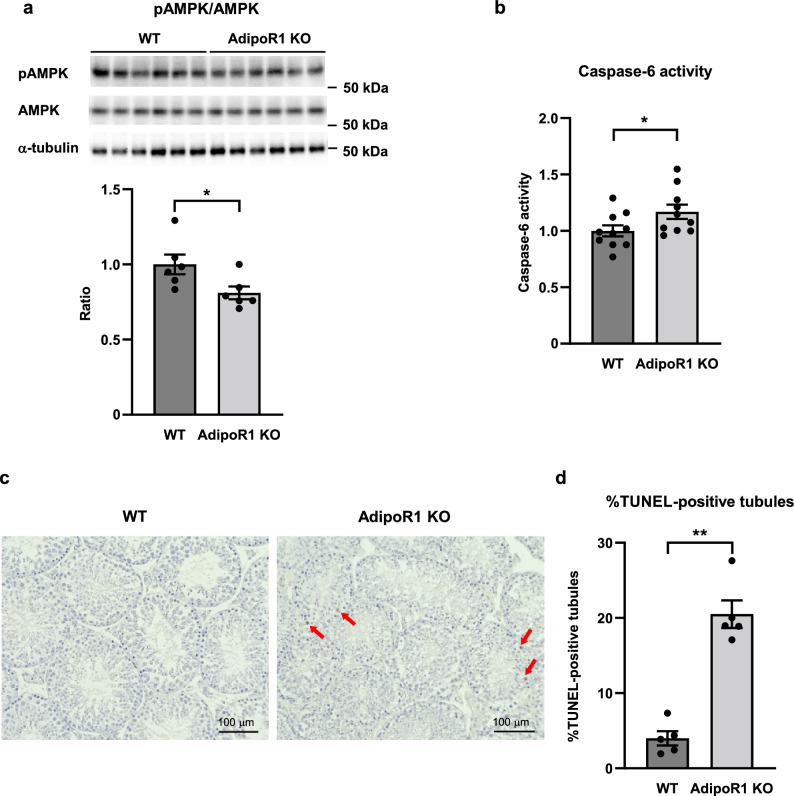
AMPK activity was decreased, and caspase-6 activity and TUNEL-positive seminiferous tubules increased, in the testis from AdipoR1 KO mice. Phosphorylation and amount of AMPK (**a**), and caspase-6 activity (**b**) in the testis from wild-type (WT) mice and AdipoR1 knockout (KO) mice. Representative micrographs from TUNEL staining of the testis from WT mice and AdipoR1 KO mice (**c**) and percentage of seminiferous tubules with TUNEL-positive cells in WT mice and AdipoR1 KO mice (**d**). Phosphorylation of AMPK were normalized to amount of AMPK. All values are presented as means ± s.e.m. *P < 0.05 and **P < 0.01 compared to WT. *P* value was determined by the unpaired two-tailed *t*-test. Scale bar, 100 μm. The red arrows indicate TUNEL-positive cells. WT, n = 6 (**a**), n = 10 (**b**), n = 5 (**d**); AdipoR1 KO, n = 6 (**a**), n = 10 (**b**), n = 5 (**d**).

Given the report that AdipoR1 activates AMP-activated protein kinase (AMPK) pathways in the liver^28^, we next focused attention on AMPK and demonstrated that the phosphorylation of AMPK was significantly suppressed in the testis from AdipoR1 KO mice compared to that in wild-type mice (Fig. 5a). Then, in light of a recent important finding that AMPK deficiency increases caspase-6 activation in the liver in nonalcoholic steatohepatitis model mice^41^, we hypothesized that the suppression of AMPK activation might induce caspase-6 activation in the testis from AdipoR1 KO mice and investigated caspase-6 activity in the testis from AdipoR1 KO mice. Interestingly, caspase-6 activity was significantly increased in the testis from AdipoR1 KO mice compared to that in wild-type mice (Fig. 5b).

Furthermore, we went on to evaluate apoptosis in the testis by TUNEL staining and demonstrated that the knockout of AdipoR1 increased the number of apoptotic seminiferous tubules (Fig. 5c,d).

### Adiponectin/AdipoR1 signaling decreased caspase-6 activity in TM4 cells

**Figure 6:**
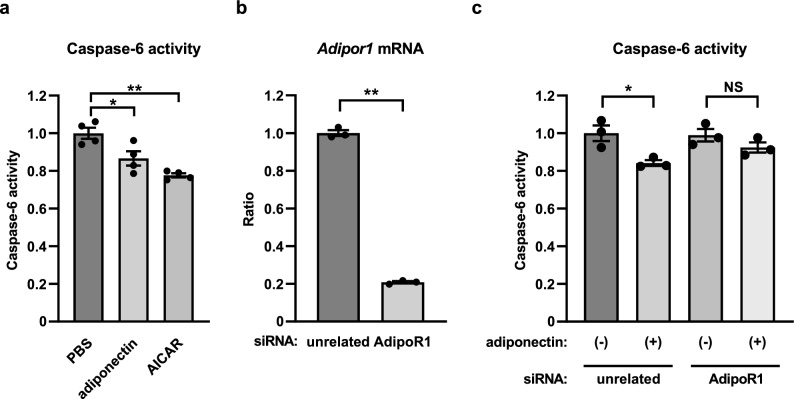
Adiponectin/AdipoR1 signaling decreased caspase-6 activity in TM4 cells. Caspase-6 activity in TM4 cells treated with 30 μg/ml adiponectin for 10 min or 2 mM acadesine (AICAR) for 60 min (**a**). *Adipor1* mRNA levels in TM4 cells transfected with the indicated specific short interfering RNA (siRNA) (**b**), and caspase-6 activity in TM4 cells transfected with the indicated siRNA and treated with 30 μg/ml adiponectin or phosphate buffered salts (PBS) for 10 min (**c**). Results of real-time PCR were normalized to *Actb*. All values are presented as means ± s.e.m. *P < 0.05 and **P < 0.01 compared to PBS, unrelated siRNA (negative control) or as indicated. *P* values were determined by the Dunnett’s multiple comparison test (**a**), unpaired two-tailed *t*-test (**b**), or Tukey’s honestly significant difference test (**c**). NS, not significant. PBS, n = 4; adiponectin, n = 4; AICAR, n = 4 (**a**). n = 3 each (**b, c**).

AdipoR1 KO mice present with hyperglycemia and insulin resistance^28^, thus suggesting the possibility that hyperglycemia and insulin resistance might contribute to increased apoptosis in the testis from the AdipoR1 KO mice. Then, we studied adiponectin/AdipoR1 signaling in vitro to investigate its direct effect. In TM4 cells incubated with 30 μg/ml adiponectin and 2 mM acadesine (AICAR), caspase-6 activity was significantly decreased (Fig. 6a). Under suppression of AdipoR1 expression with a specific short interfering RNA (siRNA) (Fig. 6b), however, no significant difference was noted in caspase-6 activity between TM4 cells incubated or unincubated with adiponectin (Fig. 6c). These experiments combined to show that adiponectin significantly reduced caspase-6 activity via AdipoR1.

### AdipoRon improved sperm motility, and decreased caspase-6 activity and TUNEL-positive seminiferous tubules in the testis from mice fed a high-fat diet

**Figure 7:**
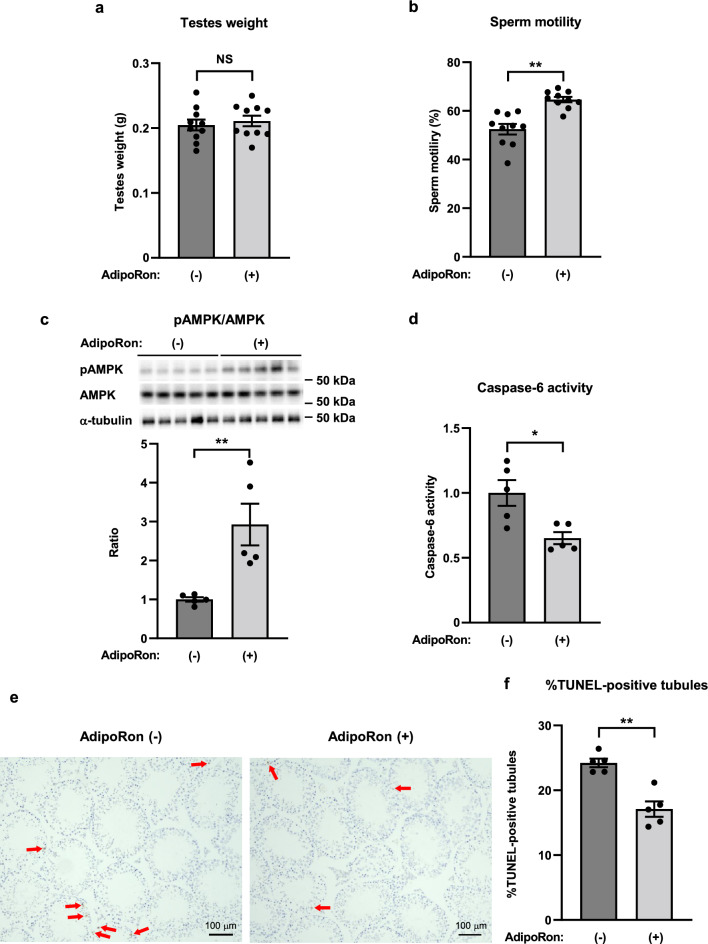
AdipoRon improved sperm motility, and decreased caspase-6 activity and TUNEL-positive seminiferous tubules in the testis from high-fat diet-fed mice. Testes weight (**a**), sperm motility (**b**), phosphorylation and amount of AMPK (**c**), caspase-6 activity in the testis (**d**), representative micrographs from TUNEL staining of the testis (**e**), and percentage of seminiferous tubules with TUNEL-positive cells (**f**), in mice on a high-fat diet, treated once daily with oral administration of AdipoRon (50 mg per kg body weight) for two weeks. Phosphorylation of AMPK were normalized to amount of AMPK. All values are presented as means ± s.e.m. *P < 0.05 and **P < 0.01 compared to control mice. *P* values were determined by the unpaired two-tailed* t*-test. NS, not significant. Scale bar, 100 μm. The red arrows indicate TUNEL-positive cells. AdipoRon (−), n = 10 (**a, b**), n = 5 (**c, d, f**); AdipoRon (+), n = 10 (**a, b**), n = 5 (**c, d, f**).

Our research group previously showed that the orally active AdipoR agonist AdipoRon activates AMPK and PPAR-α pathways^22,29^, ameliorates insulin resistance and glucose intolerance^22,29^, and reverses life shortening in obese diabetic mice^29^. Based on evidence that adiponectin/AdipoR1 signaling decreases caspase-6 activity in TM4 cells, we next studied the effects of AdipoRon on sperm parameters and apoptosis in the testis from mice fed a high-fat diet. Interestingly, oral administration of AdipoRon (50 mg per kg body weight) for 14 days had no effect on testes weight (Fig. 7a), however, AdipoRon improved sperm motility (Fig. 7b), activated AMPK in the testis (Fig. 7c), and decreased caspase-6 activity in the testis (Fig. 7d) and TUNEL-positive seminiferous tubules (Fig. 7e,f).

## Discussion

In this study we showed that decreased adiponectin/AdipoR1 signaling is implicated in obesity-induced male infertility and demonstrated for the first time that decreased adiponectin/AdipoR1 signaling involves an increase in caspase-6 activity via the AMPK-caspase-6 axis, thus resulting in suppressed spermatogenesis.

AMPK directly phosphorylates caspase-6 to inhibit its cleavage and activation^41^, thus suppressing apoptosis^41^. Caspase-6 has been suggested as an important target in Alzheimer’s disease^42,43^ as well as in nonalcoholic steatohepatitis^41^, both of which are characterized by decreased AMPK activity^41,44^. Indeed, it is shown that caspase-6 mediates a feedforward loop to sustain the caspase cascade, where increased activity of upstream executioner caspases, such as caspase-9, -3 and -7, activate the downstream executioner caspase-6, and activated caspase-6 cleaves BH3 interacting-domain death agonist (Bid) and increases mitochondrial cytochrome c release, and then increased cytochrome c release activates caspase-9 in the intrinsic pathway^41^.

The activity of AMPK is regulated by multiple factors, such as nutrients, exercise, hormones and cytokines in physiological or pathological conditions^45,46^. Our research group previously demonstrated that adiponectin/AdipoR1 signaling activates AMPK in the liver^20,25,28^ and the skeletal muscle^30^. AdipoR1 is relatively ubiquitously expressed throughout the body but expressed abundantly in the skeletal muscle, whereas AdipoR2 is found to be predominantly expressed in the liver^46^. Moreover, our research group has shown that adiponectin/AdipoR signaling is decreased in obesity, and decreased adiponectin/AdipoR signaling accounts in part for metabolic syndrome or type 2 diabetes^28,29^. In this study, we showed that testicular adiponectin/AdipoR1 signaling was decreased in obesity. Moreover, we demonstrated that decreased AMPK activity and increased caspase-6 activity in the testis from AdipoR1 KO mice and that AdipoR1 KO mice were associated with male infertility due to smaller testis, lower sperm counts and lower sperm motility. Our study showed that decreased adiponectin/AdipoR1 signaling accounts for male infertility as it involves apoptosis via the AMPK-caspase-6 axis.

Several reports have shown protective effects of adiponectin on the testis in diabetes model mice^39,40^. In streptozotocin-induced diabetic mice, it was shown that sperm parameters and the protein expression of autophagy were decreased, and the testicular endoplasmic reticulum stress and oxidative stress increased, while recombinant adiponectin treatment reversed these changes^40^. Another research group reported that administration of adiponectin elevated serum testosterone, the expression of testicular steroidogenic marker proteins, insulin receptor, and glucose transporter 8, and intra-testicular concentrations of glucose and lactate and activity of lactate dehydrogenase and antioxidant enzymes in high-fat diet-/streptozotocin-induced diabetic mice, compared to untreated diabetic mice^39^. These findings suggest that adiponectin improves testicular function by increasing the transport of glucose and lactate as well as by reducing oxidative stress. Thus, alongside these, in this study, another mechanism by which adiponectin/AdipoR1 signaling influences testicular functions has been demonstrated for the first time.

Importantly, given that decreased AdipoR expression is a hallmark of obesity^27^, establishing ways to activate adiponectin/AdipoR signal is expected to be the key for definitive treatment of obesity-related diseases. Against this background, small-molecule AdipoR agonists are attracting attention as another potential class of drugs of interest for obesity-related diseases including type 2 diabetes^47,48^. An orally active synthetic small-molecule AdipoR agonist identified by screening a library of candidate compounds^29^, AdipoRon is shown to activate AMPK and PPAR-α pathways^22,29^, ameliorate insulin resistance and glucose intolerance^22,29^, and reverse life shortening in obese diabetic mice^29^. Moreover, it was recently reported that a PEGylated AdipoRon derivative was more effective in reducing ceramides and dihydroceramides in the liver from mice fed a high-fat diet than AdipoRon^49^. In this study, we demonstrated for the first time that AdipoRon ameliorated sperm motility and apoptosis in the testis from mice fed a high-fat diet. Our data suggested that activating adiponectin/AdipoR1 signaling might be one of the therapeutic targets for obesity-induced male infertility. Given that, based on the structural information of AdipoRs^21^, much progress has been made to date not only in the analysis of the co-crystal structures of AdipoRs and AdipoRon but in the development of small-molecule AdipoR agonists for clinical use^22^, expectations are mounting for AdipoR agonists as an effective therapeutics for obesity-induced male infertility.

## Methods

### Mouse studies

All procedures were carried out in accordance with relevant guidelines and regulations as approved by the Animal Care Committees of The University of Tokyo and the Institute of Medical Science, Asahi Life Foundation, and complied with the standards stated in the “Guide for the Care and Use of Laboratory Animals” (National Institutes of Health, revised 2011). The study is reported in accordance with ARRIVE guidelines. Male mice were 6–55 weeks of age at the time of the study. They were housed in cages and maintained on a 12 h light–dark cycle with access to chow and water ad libitum. In these experiments, we used normal chow diet consisting of 24.9% (wt/wt) proteins, 4.6% fibers, 7.1% ashes, 49.5% carbohydrates, 4.8% fat and 9.1% water (CE-2, CLEA Japan Inc.) or high-fat diet consisting of 25.5% (wt/wt) protein, 2.9% fibers, 4.0% ashes, 29.4% carbohydrates, 32.0% fat and 6.2% water (High Fat Diet 32, CLEA Japan Inc.). High-fat diet was fed to male mice from 6 weeks old onwards. C57Bl/6 mice were purchased from Charles River Laboratories Japan, Inc. and Japan SLC, Inc. Akita mice were purchased from Japan SLC, Inc.

### Generation of AdipoR1 KO mice

*Adipor1*^−/−^ mice (C57Bl/6 background) were generated as described previously^28^. All experiments in this study were conducted on male littermates.

### Administration of AdipoRon

For administration, AdipoRon (Combi-Blocks, #QV-9395) was prepared in 0.5% methyl cellulose (WAKO, #133-17815). AdipoRon (50 mg per kg body weight) or 0.5% methylcellulose was orally administrated to high-fat diet-fed mice from 8 weeks of age once daily for two weeks. The sampling of the mice parameters was performed at 10 weeks of age.

### Mating assay

Male C57Bl/6 mice fed a high-fat diet for 28 weeks, AdipoR1 KO mice fed standard chow diet, Akita mice fed standard chow diet, and age matched control mice fed standard chow diet were used in a mating assay. Each male mouse was caged with three or four female C57Bl/6 mice for consecutive five days. The percentage of mice achieving a pregnancy were calculated for each male.

### Testis and semen analysis in mice

Testis and epididymides were carefully dissected. Bilateral testes were weighed and frozen in liquid nitrogen, and then processed for real-time PCR, western blot analysis or caspase-6 activity assay. Separated cauda epididymis from each mouse was immediately placed into modified HTF medium with HEPES (KITAZATO, #93421) at room temperature. The sperm number in the suspension was counted using a hemocytometer on a Nikon DIAPHOT 300 microscope at  200× magnification. At least 200 sperm were counted in each sperm sample. We assessed motility rate as the percentage of the sum of sperms with progressive motility and non-progressive motility per total sperm number.

### Real-time PCR

Real-time PCR was performed according to the method described previously^22,28–30^. Total RNA was prepared from whole testis or TM4 cells with ISOGEN (Nippon Gene, #311-02501), according to the manufacturer’s instructions. We used the real-time PCR method to quantify mRNAs^20^, with slight modification. The real-time PCR was performed using specific TaqMan Gene Expression Assays (Thermo Fisher Scientific) for *Adipor1* (Mm01291334_mH), *Adipor2* (Mm01184029_m1), *Bax* (Mm00432051_m1), *Casp3* (Mm01195085_m1), *Casp6* (Mm01321726_g1), *Casp9* (Mm00516563_m1), *Rn18s* (Mm03928990_g1) and *Actb* (Mm00607939_s1). The primers for real-time PCR are shown in Table S1.

### Histology and TUNEL staining

For histological examination, each whole testis was fixed in Super Fix (Kurabo, #KY-500) at 4 °C overnight, washed and then was paraffin-embedded and sectioned. Sections were stained with hematoxylin and eosin, and In Situ Cell Death Detection Kit, POD (Roche, #11684817910) following the provided protocol for apoptosis detection. Six to eight micrographs from all regions of the testis were captured for analysis. Seminiferous tubules were evaluated by a trained pathologist for morphometry. At least 90 tubules from all testicular regions were counted for each sample to quantify its apoptotic seminiferous tubules.

### Western blot analysis

The whole mouse testis was homogenized in cold RIPA buffer (Cell Signaling Technology, #9806) containing 1 mM benzylsulfonyl fluoride (PMSF) (WAKO, #164-12181) and protease inhibitor cocktail (Complete EDTA-free, Roche, #11873580001). Lysates were centrifuged at 15,000 rpm for 15 min at 4 °C and supernatants were used for western blot analysis. Western blot analyses were performed with anti-phosphorylated AMPK (Cell Signaling technology, 1:1000; #2535), anti-αAMPK (Cell Signaling technology, 1:1000; #2532), anti-AdipoR1 (Immuno-Biological Laboratories, 1:1000; #18993), anti-cleaved caspase-3 (Cell Signaling technology, 1:1000; #9661), anti-cleaved caspase-6 (Cell Signaling technology, 1:1000; #9761), anti-cleaved caspase-9 (Cell Signaling technology, 1:1000; #9509), anti-α-tubulin (Cell Signaling technology, 1:4000; #2125) and HRP-linked anti-rabbit IgG (Cell Signaling technology, 1:2000–1:20000; #7074) antibodies. Uncropped western blot images are shown in Fig. S2-S5.

### Studies with TM4 cells

The mouse Sertoli cell-line TM4 was purchased from American Type Culture Collection (ATCC, #CRL-1715). TM4 cells were cultured in a 1:1 mixture of Ham'S F12 medium and Dulbecco's modified Eagle's medium with 1.2 g/L sodium bicarbonate and 15 mM HEPES (D-MEM/Ham's F-12) (WAKO, #042-30555) containing 5% horse serum (Invitrogen, #26050-088), 2.5% fetal bovine serum (FBS) (Bio-West, #S1600-500), and 10% penicillin/streptomycin (WAKO, #168-23191) at 37 °C in a humidified incubator with 5% CO_2_. TM4 cells were seeded in 100 mm cell culture dishes and 12-well plates coated with Cellmatrix Type I-c (Nitta gelatin, #631-00771). Three days later, TM4 cells were incubated with D-MEM/Ham's F-12 without horse serum, FBS or penicillin/streptomysin for 10 h, then treated with either 2 mM acadesine (AICAR) (Adipogen Life Sciences, #AG-CR1-0061-M010) for 60 min, 30 μg/ml adiponectin (Enzo, #ALX-522-063-C050) for 10 min, or phosphate buffered salts (PBS) (TaKaRa, #T900) for 10 min. TM4 cells were transfected by using Lipofectamine RNAiMAX Transfection Reagent (Invitrogen, #13778) and following the manufacturer’s instructions.

### RNA interference

Silencer Select Pre-Designed siRNA (Thermo Fisher Scientific) for *Adipor1* (s91210) or Silencer Select Negative Control No.1 siRNA (#4390843) were transfected into TM4 cells by using Lipofectamine RNAiMAX Transfection Reagent. Forty-two hours after transfection, TM4 cells were incubated with D-MEM/Ham's F-12 without horse serum, FBS or penicillin/streptomycin for 10 h, then treated with 30 μg/ml adiponectin for 10 min, or PBS for 10 min, and then the cells were lysed for caspase-6 activity assay. Transfection efficacy was monitored based on gene expression levels of *Adipor1* using the real-time PCR. The siRNA sequence for downregulating AdipoR1 is shown in Table S2.

### Caspase-6 activity assay

The testis or cell was homogenized with lysis buffer. Capase-6 activity was determined with Caspase-6 Assay Kit (Colorimetric) (Abcam, #ab39709), according to manufacturer’s instruction. Caspase-6 activity was normalized to total protein amount.

### Statistical analysis

Results are expressed as mean ± s.e.m. Differences between two groups were assessed for significance using the unpaired two-tailed *t*-test. Data involving more than two groups were assessed by analysis of variance (ANOVA) followed by the Dunnett’s multiple comparison test or Tukey’s honestly significant difference test. Representative data from one of 2–5 independent experiments are shown. Every experiment was performed several times with essentially the same results.

### Supplementary Information

Supplementary Information 1.

Supplementary Information 2.

## Data Availability

The source data for the figures are available in Supplementary Data 1. Uncropped images of western blots are shown in Supplementary Fig. 2-5. All other data that support the findings of this study are available from the corresponding authors on reasonable request.
