# Supplementary material for: Decreased AdipoR1 signaling and its implications for obesity-induced male infertility
Source: Sci Rep. 2024 Mar 8;14:5701. doi: 10.1038/s41598-024-56290-0 (PMC10923778; doi:10.1038/s41598-024-56290-0)
Supplement: Supplementary file 2 — Supplementary Information 2. [file 41598_2024_56290_MOESM2_ESM.docx]

**Decreased AdipoR1 signaling and its implications for obesity-induced male infertility**

Toshiko Kobori^1^, Masato Iwabu^2,3^*, Miki Okada-Iwabu^3,4^*, Nozomi Ohuchi^3^, Akiko Kikuchi^1^, Naoko Yamauchi^5^, Takashi Kadowaki^3,6^, Toshimasa Yamauchi^3^, Masato Kasuga^1^

1 *Division of Diabetes and Metabolism, The Institute of Medical Science, Asahi Life Foundation; Chuo-ku, Tokyo, 103-0002, Japan.*

2 *Department of Endocrinology, Metabolism and Nephrology, Graduate School of Medicine, Nippon Medical School; Bunkyo-ku, Tokyo, 113-8603, Japan.*

3 *Department of Diabetes and Metabolic Diseases, Graduate School of Medicine, The University of Tokyo; Bunkyo-ku, Tokyo, 113-8655, Japan.*

4 *Laboratory for Advanced Research on Pathophysiology of Metabolic Diseases, The University of Tokyo; Bunkyo-ku, Tokyo, 113-8655, Japan.*

5 *Digital Pathology Center, Asahi General Hospital; Asahi-shi, Chiba, 289-2511, Japan.*

6 *Toranomon hospital; Minato-ku, Tokyo, 105-8470, Japan.*

*Corresponding authors. Email: m-iwabu@nms.ac.jp (M.I.) or omiki@m.u-tokyo.ac.jp (M.O.-I.)

**Supplementary Information**

**Supplementary table**

**Table S1. The primers for real-time PCR**

|  | TaqMan Gene Expression Assays ID | probe context sequence |
| --- | --- | --- |
| *Adipor1* | Mm01291334_mH | GGACACATCTGCTTGGTTTTGTGCT |
| *Adipor2* | Mm01184029_m1 | AGTTTGTTTGTAAGGTGTGGGAAGG |
| *Bax* | Mm00432051_m1 | CAAGACCAGGGTGGCTGGGAAGGCC |
| *Casp3* | Mm01195085_m1 | TCTACAGCACCTGGTTACTATTCCT |
| *Casp6* | Mm01321726_g1 | TACAAAAGTAGGGAAGTGTTCGATC |
| *Casp9* | Mm00516563_m1 | AGGATATTCAGCAGGCAGGATCTGG |
| *Rn18s* | Mm03928990_g1 | TACTTGGATAACTGTGGTAATTCTA |
| *Actb* | Mm00607939_s1 | ACTGAGCTGCGTTTTACACCCTTTC |

**Table S2. The siRNA sequence**

|  | Silencer Select Pre-Designed siRNA  ID | sense siRNA sequence |
| --- | --- | --- |
| *Adipor1* | s91210 | AGACCAAAUAUGUACUUCA |

**Supplementary figure**

**Supplementary figure 1**

**
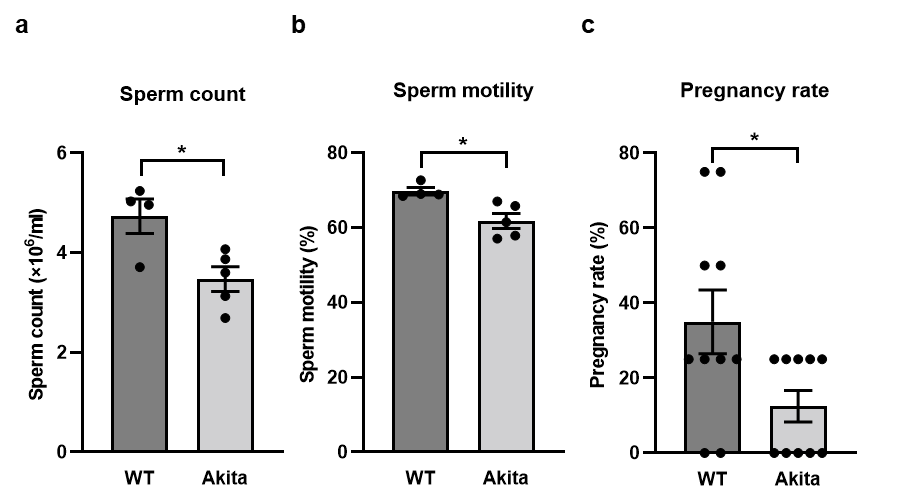
**

**Fig. S1.** **Sperm count, sperm motility and sperm fertilizing ability in male model mice of type 1 diabetes were decreased.**

Sperm count (**a**), sperm motility (**b**) and pregnancy rate (**c**) in wild-type (WT) mice and model mice of type 1 diabetes (Akita). All values are presented as means ± s.e.m. **P* < 0.05 compared to WT. *P* values were determined by the unpaired two-tailed *t*-test. WT, n = 4 (**a, b**), n = 10 (**c**); Akita, n = 5 (**a, b**), n = 10 (**c**).

**Supplementary figure 2**


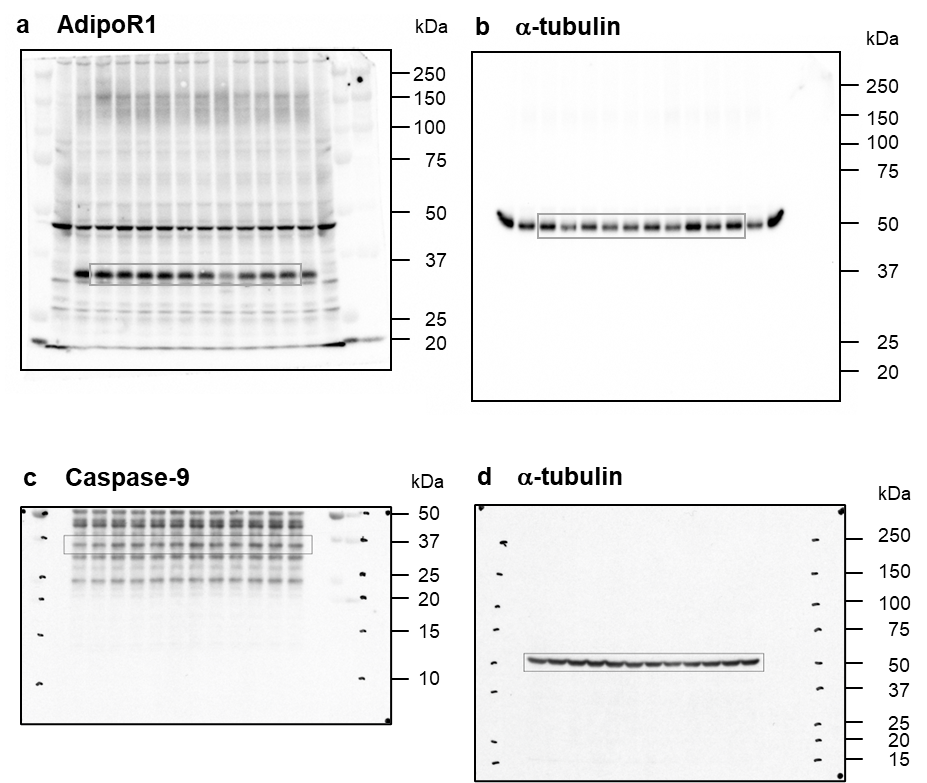


**Fig. S2. Raw western blots data in Fig. 2**

Uncropped images of western blots in Fig. 2b. AdipoR1 (**a**) and α-tubulin (**b**). Uncropped images of western blots in Fig. 2f. Caspase-9 (**c**) and α-tubulin (**d**).

**Supplementary figure 3**

**
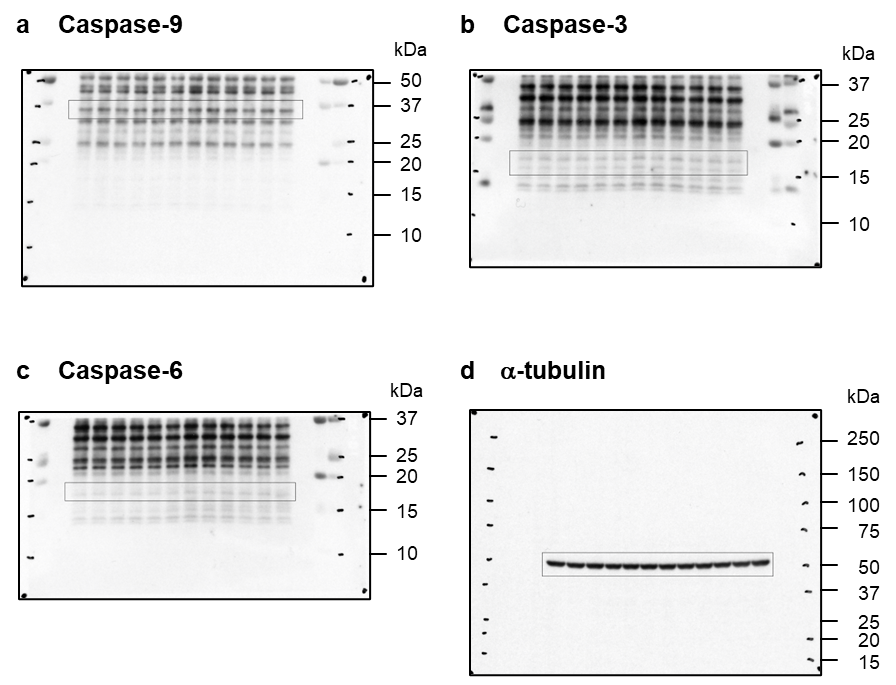
**

**Fig. S3. Raw western blots data in Fig. 4**

Uncropped images of western blots in Fig. 4d. Caspase-9 (**a**), caspase-3 (**b**), caspase-6 (**c**) and α-tubulin (**d**).

**Supplementary figure 4**

**
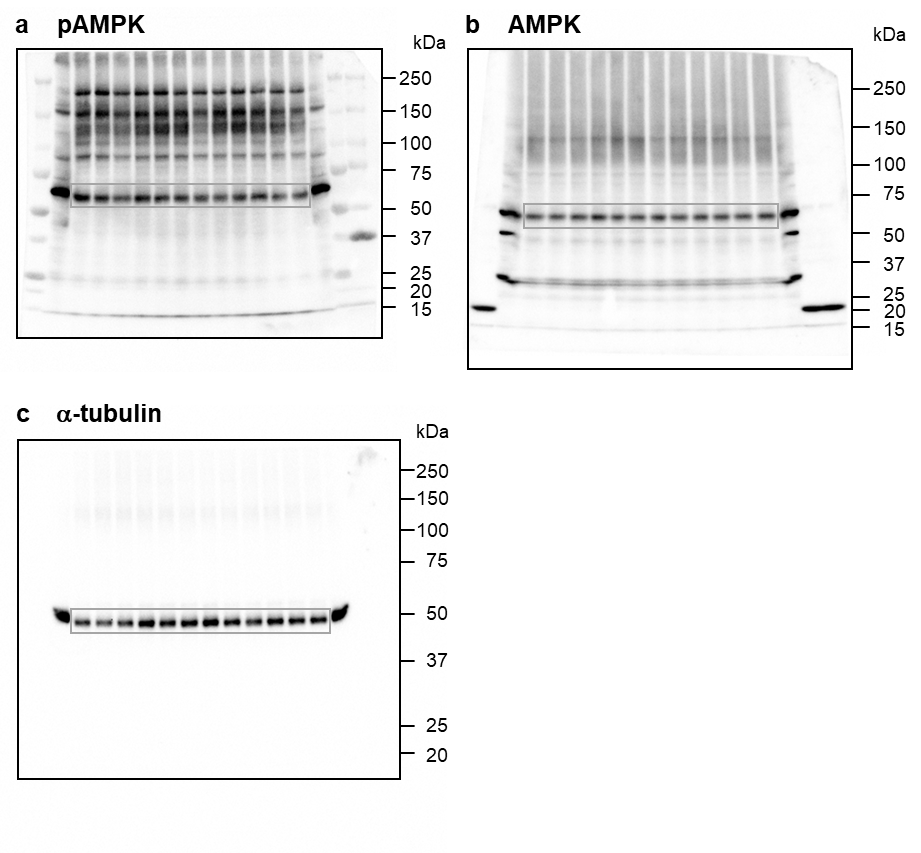
**

**Fig. S4. Raw western blots data in Fig. 5**

Uncropped images of western blots in Fig. 5a. pAMPK (**a**), AMPK (**b**) and α-tubulin (**c**).

**Supplementary figure 5**

**
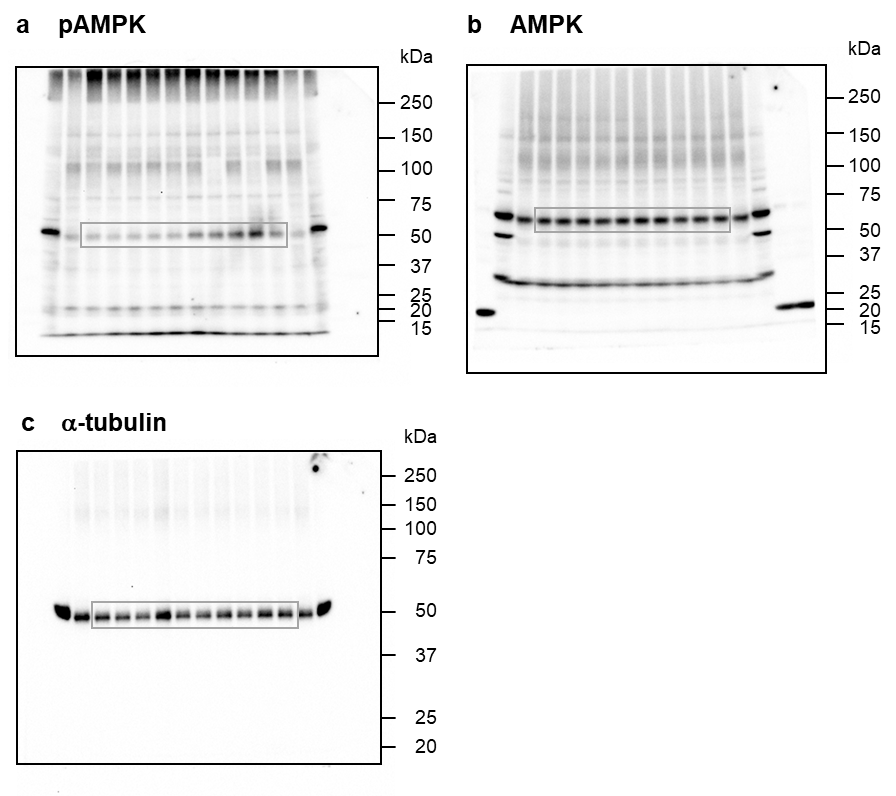
**

**Fig. S5. Raw western blots data in Fig. 7**

Uncropped images of western blots in Fig. 7c. pAMPK (**a**), AMPK (**b**) and α-tubulin (**c**).
